# Identification of RNA-Binding Proteins with Prognostic Prediction in Colorectal Cancer

**DOI:** 10.1155/2021/7114445

**Published:** 2021-11-05

**Authors:** Xiao-fen Bai, Jing-wen Liu

**Affiliations:** ^1^Department of Gastroenterology, Guangzhou Red Cross Hospital, Jinan University, Guangzhou, Guangdong, China; ^2^Endoscopy Center, The First Affiliated Hospital, Jinan University, Guangzhou, Guangdong, China

## Abstract

Colorectal cancer (CRC) is one of the most common malignancies of the digestive system. Recent studies have revealed the importance of RNA-binding proteins (RBPs) in tumorigenesis, but their role in CRC remains unclear. The present study systematically analyzed the relationships between RBPs and CRC using data from The Cancer Genome Atlas. We detected 483 differentially expressed RBPs and identified a series of pathways and processes using GO (Gene Ontology) analysis and KEGG (Kyoto Encyclopedia of Genes and Genomes) pathway analysis. Analyzing protein–protein interactions and modules identified the edges and modules of RBPs. Univariate and multivariate Cox regression analyses were then used to construct a prognostic model that included 13 RBPs. Survival analyses indicated that the overall survival (OS) was significantly lower for CRC patients in the high-risk group than for those in the low-risk group, and that high risk scores were associated with poor OS. Finally, we constructed a nomogram that included 13 RBPs for calculating the estimated survival probabilities of CRC patients at 1, 2, and 3 years. Calibration plots indicated good conformity between the predicted and observed outcomes. This study has revealed that the expression of RBPs differs between CRC and normal tissues. A prognostic model based on 13 RBP coding genes has been developed that can provide independent prognoses of CRC.

## 1. Introduction

Colorectal cancer (CRC) is one of the most common malignancies of the digestive system [1]. Recent changes in diet characteristics, population aging, and living standards have resulted in gradual increases in the incidence and mortality of CRC in China [2]. There were 388,000 new CRC cases and 187,000 deaths from CRC in China in 2015, and the main treatments for CRC are currently surgery, radiotherapy, chemotherapy, and other therapies [2]. The molecular mechanisms underlying CRC are still not fully understood, indicating the need for further searches of novel gene targets.

RNA-binding proteins (RBPs) perform various functions to maintain cellular homeostasis. These proteins play vital roles in regulating numerous essential cellular processes, including RNA splicing, modification, transport, localization, stability, degradation, and translation [3, 4]. RBPs can interact with various classes of RNAs, including mRNAs, tRNAs, snRNAs, snoRNAs, and ncRNAs [5]. It has been estimated that more than 1,500 proteins can bind RNA in the human genome [5]. Any significant change or disturbance in the RBPs regulating these essential functions can lead to diseases, including cancer [4, 6].

Recent studies have revealed the importance of RBPs in tumorigenesis, with altered expression, localization, or posttranslational modification of RBPs contributing to tumorigenesis by increasing the expression of oncogenes and decreasing the expression of tumor suppressor genes [7]. These observations suggest that regulating the expression levels of RBPs could represent a novel approach to treating tumors. RBPs have already been shown to play an essential role in gastrointestinal tumors such as esophageal cancer and gastric cancer [3], but their role in CRC has remained unclear.

## 2. Materials and Methods

### 2.1. Differentially Expressed RBPs

We downloaded CRC data from The Cancer Genome Atlas (TCGA), including RNA-seq data of CRC tissues and normal colorectal tissues, the clinicopathological parameters, and prognostic information. Differentially expressed RBPs were identified using the limma package in *R* (version4.0.0) based on a false-discovery rate (FDR) of <0.05 and a log2fold change (logFC) of ≥0.5.

### 2.2. GO Analysis and KEGG Pathway Analysis

We performed GO (Gene Ontology) analysis and KEGG (Kyoto Encyclopedia of Genes and Genomes) pathway analysis with differentially expressed RBPs to identify related processes and pathways using *R* (version4.0.0). Probability values of *P* < 0.05 were considered statistically significant.

### 2.3. Protein–Protein Interactions and Module Screening

STRING (version10) and Cytoscape (version3.6.1) software was used to construct a protein–protein interaction (PPI) network among all differentially expressed RBPs. The key modules were screened from the PPI network with scores of >7 and more than five nodes using the MCODE (Molecular Complex Detection) plug-in in Cytoscape.

### 2.4. Prognostic Model Construction and Evaluation

The survival package in *R* (version4.0.0) was used to perform univariate and multivariate Cox regression analyses with the differentially expressed RBPs to identify the genes related to prognosis. We then randomly divided the CRC patients into training and testing cohorts. The risk score for each CRC patient was calculated to evaluate the performance of the prognostic model, and these scores were then used to divide the patients into low-risk and high-risk subgroups based on the median risk score. Kaplan-Meier survival curves were constructed, and the log-rank test was used to determine how the overall survival (OS) differed between the two risk subgroups in the training and testing cohorts. The survivalROC package was used to construct receiver operating characteristics (ROC) curves for predicting the performance of the model, and finally, a nomogram was produced.

## 3. Results

### 3.1. Identification of Differentially Expressed RBPs

**Figure 1:**
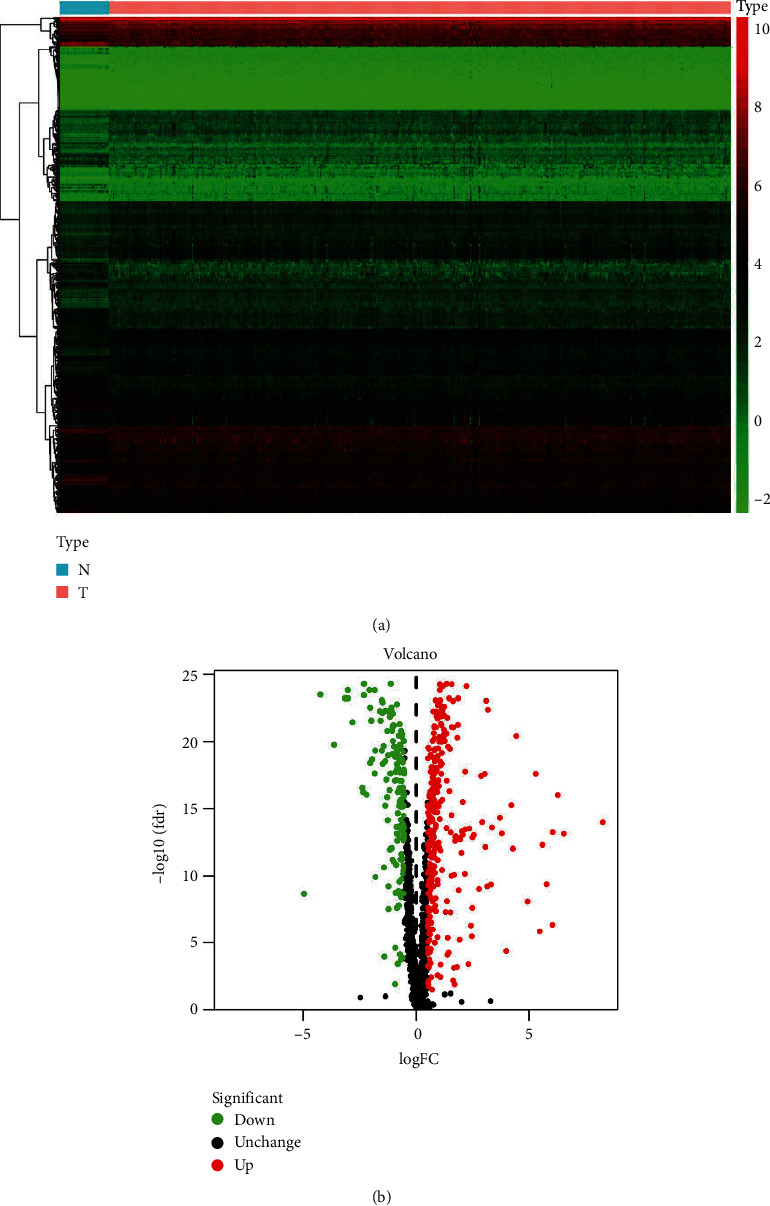
The expression of differentially expressed RBPs. (a) The heat map of differentially expressed RBPs in colorectal cancer and normal colorectal samples. (b) The volcano plot of differentially expressed RBPs.

We downloaded RNA sequencing data and clinicopathological parameters of CRC from the TCGA, which comprised 568 CRC samples and 44 normal colorectal samples. Our analysis of 1542 RBPs [8] revealed that 483 met our inclusion criteria of FDR < 0.05 and logFC ≥ 0.5: 161 were downregulated RBPs, and 322 were upregulated RBPs. The expression of these genes is shown in Figure 1.

### 3.2. Results of GO Analysis and KEGG Pathway Analysis

**Figure 2:**
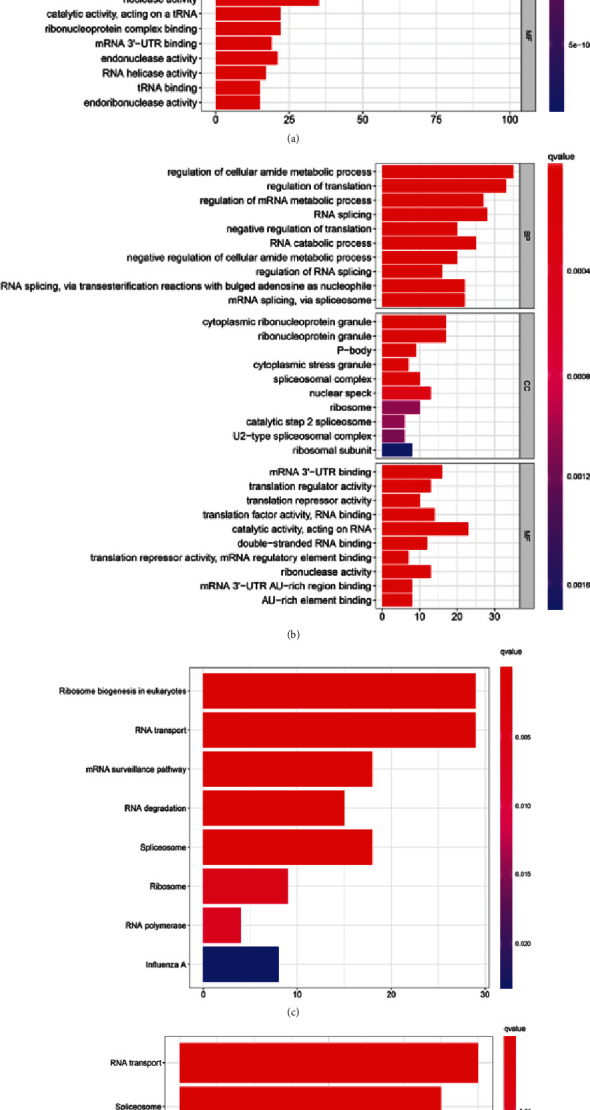
Functional annotations of the differentially expressed RBPs in CRC. (a, b) GO analysis. (c, d) KEGG pathway analysis.

We divided the differentially expressed RBPs into upregulated and downregulated groups based on their expression levels, and then GO analysis was used to identify meaningful processes. As for biological processes, upregulated genes were significantly associated with ncRNA processing, ribosome biogenesis, rRNA metabolic process, rRNA processing, and RNA phosphodiester bond hydrolysis. Downregulated genes were significantly associated with cellular amide metabolic process, regulation of translation, regulation of mRNA metabolic process, RNA splicing, and negative regulation of translation. Cellular components showed that upregulated genes were significantly associated with per ribosome, small subunit processome, cytoplasmic ribonucleoprotein granule, nucleolar part, and ribonucleoprotein granule, while downregulated genes were associated with cytoplasmic ribonucleoprotein granule, ribonucleoprotein granule, P-body, cytoplasmic stress granule, and spliceosomal complex. Regarding molecular functions, upregulated genes were significantly associated with catalytic activity, acting on RNA, ribonuclease activity, nuclease activity, catalytic activity, acting on a tRNA, and ribonucleoprotein complex binding, whereas downregulated genes were involved in mRNA 3′-UTR binding, translation regulator activity, translation repressor activity, translation factor activity, RNA binding, catalytic activity, and acting on RNA (Figures 2(a) and 2(b)).

KEGG pathway analysis suggested that the upregulated genes were mainly involved in the following eight pathways (*P* < 0.05): ribosome biogenesis in eukaryotes, RNA transport, mRNA surveillance pathway, RNA degradation, spliceosome, ribosome, RNA polymerase, and influenza A. Meanwhile, downregulated genes were associated with RNA transport, spliceosome, TGF-beta signaling pathway, hepatitis C, ribosome, influenza A, and RNA degradation. These results are shown in Figures 2(c) and 2(d).

### 3.3. PPI and Module Analysis

**Figure 3:**
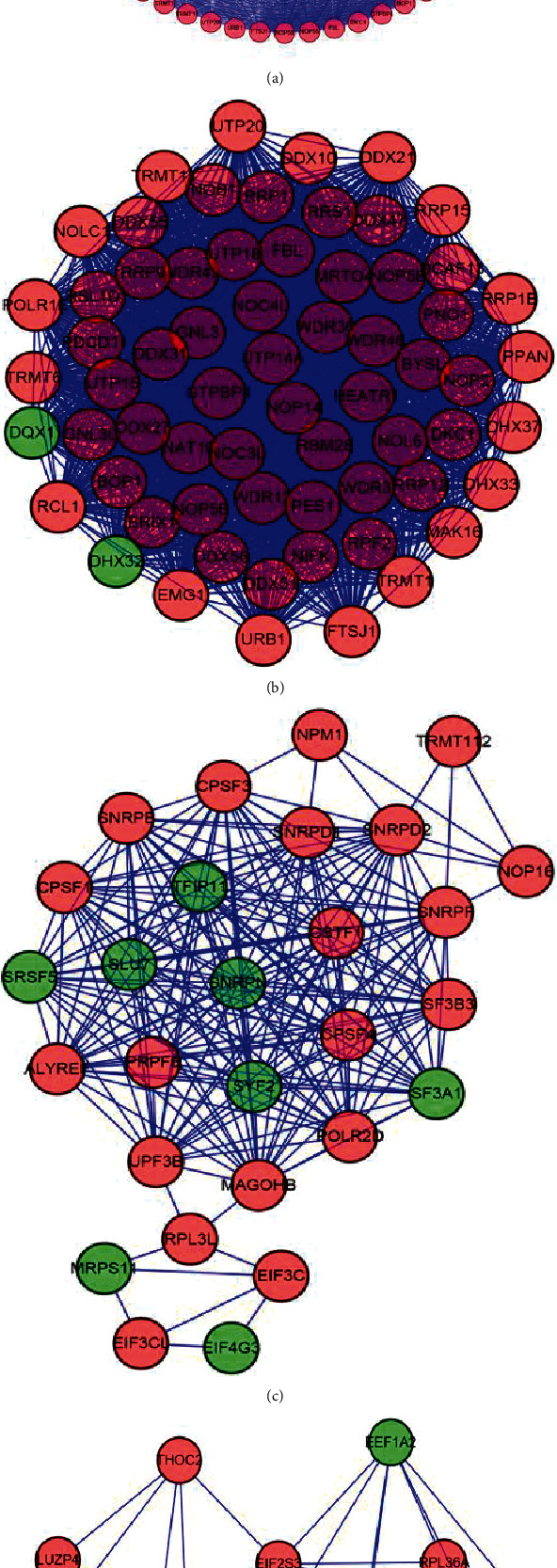
PPI network and module analysis. (a) PPI network of 3 critical modules. (b) Critical module 1 in PPI network. (c) Critical module 2 in the PPI network.

STRING software was used to construct the PPI network, which comprised 456 nodes and 6831 edges (combined score > 0.4). We further analyzed the coexpression network to identify the following top 3 significant modules using the MCODE plug-in in Cytoscape: module 1 consisted of 65 nodes and 1995 edges, module2 consisted of 28 nodes and 209 edges, and module 3 consisted of 16 nodes and 60 edges. These three modules are shown in Figure 3.

The GO analysis showed that the RBPs in module 1 were significantly associated with ribosome biogenesis, rRNA metabolic process, rRNA processing, ncRNA processing, and per ribosome. The RBPs in module 2 were associated with RNA splicing, via transesterification reactions with bulged adenosine as nucleophile, mRNA splicing, via spliceosome, RNA splicing, via transesterification reactions, RNA splicing, and catalytic step 2 spliceosome. The RBPs in module 3 were involved in translation factor activity, RNA binding, cytoplasmic translational initiation, translational initiation, cytoplasmic translation, and translation initiation factor activity.

The KEGG pathway analysis showed that the RBPs in module 1 were significantly associated with ribosome biogenesis in eukaryotes. The RBPs in module 2 were significantly associated with spliceosome, mRNA surveillance pathway, and RNA transport, while those in module 3 were significantly associated with RNA transport, legionellosis, leishmaniasis, spliceosome, and ribosome. These results are presented in Table 1.

### 3.4. Construction and Verification of the Prognostic Model

**Figure 4:**
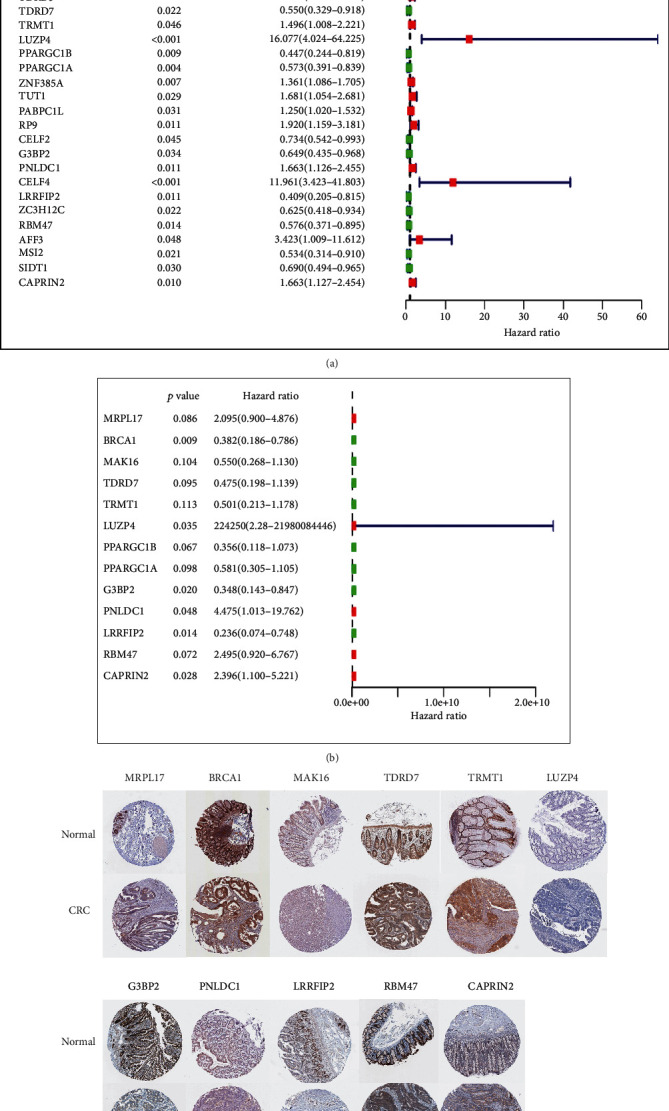
The Cox regression and protein expression of hub genes.(a) Univariate Cox regression of differentially expressed RBPs related to prognosis. (b) Multivariate Cox regression of differentially expressed RBPs to build a prognostic model. (c) Validation of protein expression of hub genes in normal colorectal tissue and CRC using the HPA database.

Univariate Cox regression confirmed that 31 of the 483 differentially expressed RBPs were related to the prognosis of CRC (Figure 4(a)). After randomly dividing the CRC patients into the training and testing cohorts, multivariate Cox regression identified the following 13 RBPs in the training cohort for constructing a prognostic model: MRPL17, BRCA1, MAK16, TDRD7, TRMT1, LUZP4, PPARGC1B, PPARGC1A, G3BP2, PNLDC1, LRRFIP2, RBM47, and CAPRIN2 (Figure 4(b)). We used data from the Human Protein Atlas database to analyze immunohistochemistry results.  Figure 4(c) shows that the protein expression of CAPRIN2 was higher in CRC tissue than in normal colorectal tissue, where those of BRCA1, G3BP2, LRRFIP2, and RBM47 were lower in CRC tissue.

**Figure 5:**
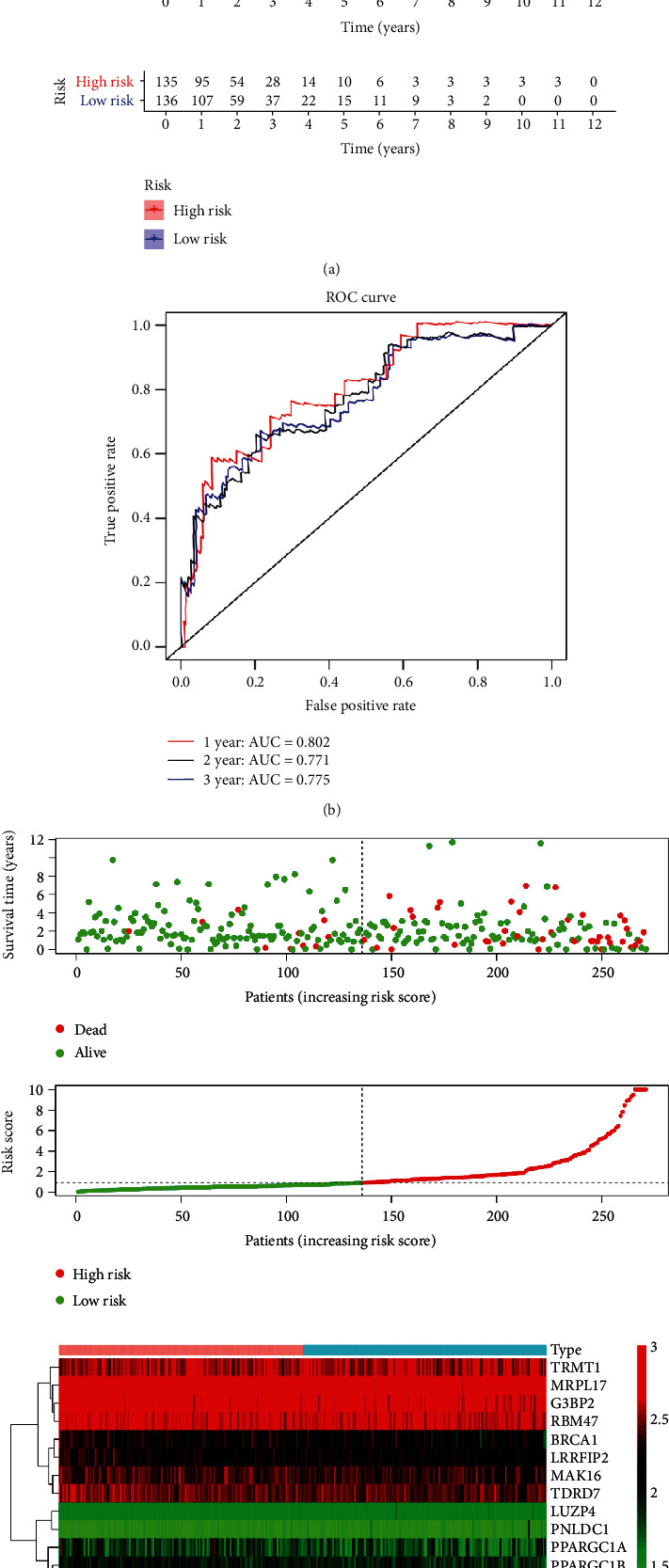
Risk score analysis of 13-RBP prognostic model in CRC. (a) Survival analysis according to risk score. (b) ROC curves. (c) Survival status and expression heat map of patients.

We calculated the risk score for each patient and divided the patients into high-risk and low-risk groups. The training and testing cohorts indicated that the OS was significantly lower among patients in the high-risk group (Figure 5(a)). ROC curves were used to further evaluate the predictive performance of the 13 genes. The areas under the ROC curves (AUCs) for 1-, 2-, and 3-year OS in the training cohort were 0.802, 0.771, and 0.775, respectively (Figure 5(b)). The survival status and an expression heat map of patients with the 13 RBPs in the subgroups were shown in Figure 5(c). This figure shows that a higher risk score did not significantly decrease the survival time but it did significantly increase the mortality rate.

**Figure 6:**
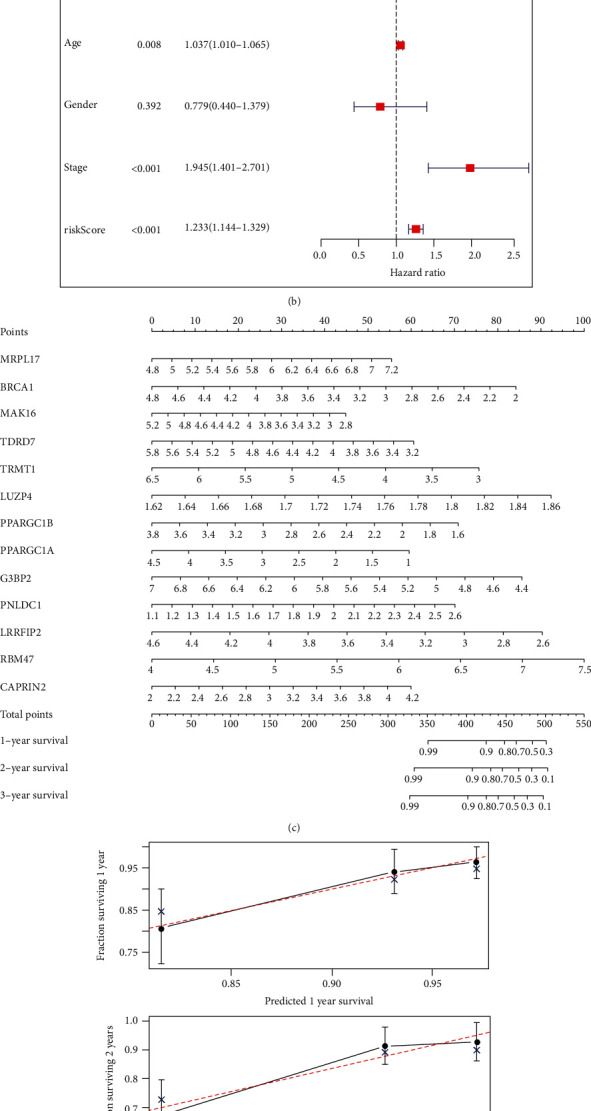
Nomogram of 13 RBPS. (a) Univariate analysis of clinicopathological parameters. (b) Multivariate analysis of clinicopathological parameters. (c) Nomogram to predict one, two, and three-year OS. (d) Calibration plots of the nomogram to predict OS at one, two, and three years.

We also assessed the prognostic value of different clinicopathological parameters in the training and testing cohorts through univariate and multivariate Cox regression analyses. The results showed that age, pathological stage, and high-risk scores were associated with poor OS (*P* < 0.01) (Figures 6(a) and 6(b)). A quantitative model for the prognosis of CRC was produced by constructing a nomogram for the 13 RBPs (Figure 6(c)). This nomogram can be used to calculate the estimated survival probabilities of CRC patients at 1, 2, and 3 years by drawing a vertical line from the axis showing the total score to each prognosis axis. We also constructed calibration plots, which indicated that there was good conformity between the predicted and observed outcomes (Figure 6(d)).

## 4. Discussion

RBP malfunction resulting in altered RNA metabolism can lead to genome-wide changes in the transcriptome and proteome of cells so as to affect their growth, proliferation, invasion, and death. It is therefore not surprising that altered expression of RBPs is a common phenomenon during the development and progression of cancers [7].

The present GO analysis shows that the differentially expressed RBPs were associated with various mechanisms including ribosome biogenesis, rRNA metabolic process, rRNA processing, ncRNA processing, and preribosome. In contrast, the KEGG pathway analysis revealed the involvement of mechanisms such as ribosome biogenesis in eukaryotes, spliceosome, mRNA surveillance pathway, RNA transport, RNA transport, legionellosis, leishmaniasis, spliceosome, and ribosome. Previous studies have revealed the RBPs related to pathways such as RNA splicing, modification, transport, localization, stability, degradation, and translation [3, 4]. The results of the present study are similar to those of previous studies, by showing that RBPs are involved in pathways related to RNA metabolism.

The 13-RBP prognostic model comprised MRPL17, BRCA1, MAK16, TDRD7, TRMT1, LUZP4, PPARGC1B, PPARGC1A, G3BP2, PNLDC1, LRRFIP2, RBM47, and CAPRIN2. Oh et al. [9] found an increased risk of CRC for BRCA1. A stratified analysis revealed that the PPARG rs3856806 C>T polymorphism also increased the risk of CRC [10]. LRRFIP2 is associated with an MLH1 mutation that affects the occurrence of Lynch syndrome [11]. A pancancer analysis of the EMT signatures identified that RBM47 was downregulated in CRC progression [12].

It have not been reported that MRPL17, MAK16, TDRD7, TRMT1, LUZP4, G3BP2, PNLDC1, and CAPRIN2 were related to CRC, and so further research is necessary. In the present study, the 1-, 2-, and 3-year AUCs of the prognostic model were 0.802, 0.771, and 0.775, respectively, indicating that this model exhibited moderate predictive performance. A nomogram was constructed to predict the probability of CRC patients surviving for 1, 2, and 3 years, and the underlying predictive models suggested that patients with high-risk scores have a poor prognosis. Risk models can be used to divide patients into high-risk and low-risk groups, allowing targeted further treatment interventions.

Li et al. [13] established a prognostic model of RBP-related genes in lung squamous cell carcinoma, which suggested that risk scores have good predictive performance. Clinical prognostic models have been widely used in medical research and practice [14]. Such prognostic model and their associated nomograms can be used by doctors and patients to make better joint decisions and by clinical researchers for more accurately screening suitable research subjects. Clinical prognostic models related to gene targets have also been studied extensively, such as m6A-related gene models [15] and an autophagy-related gene signature [16].

In summary, this study explored new prognostic indicators by screening differentially expressed RBPs, performing function predictions, and constructing prognostic models. The new prognostic indicators that have been identified provide a biological background for the clinical predictions. However, this study was subject to some limitations. The model constructed in this study involved only gene expression and did not include clinicopathological parameters. In addition, the study findings were not validated in a clinical patient cohort.

## 5. Conclusion

This study found that the expression of RBPs differed between CRC and normal tissues. A prognostic model based on 13 RBP coding genes has been developed that could act as an independent prognostic signature for CRC.

## Figures and Tables

**Table 1:** GO and KEGG pathway analysis results for differentially expressed RBPs in 3 critical modules.

MCODE	Processes or pathways	*P* value
GO		
Module 1	Ribosome biogenesis	3.60*E*-79
	rRNA metabolic process	7.92*E*-78
	rRNA processing	1.56*E*-76
	ncRNA processing	2.71*E*-73
	Preribosome	1.39*E*-57
Module 2	RNA splicing, via transesterification reactions with bulged adenosine as nucleophile	2.30*E*-28
	mRNA splicing, via spliceosome	2.30*E*-28
	RNA splicing, via transesterification reactions	2.70*E*-28
	RNA splicing	1.73*E*-26
	Catalytic step 2 spliceosome	3.34*E*-24
Module 3	Translation factor activity, RNA binding	5.06*E*-13
	Cytoplasmic translational initiation	6.34*E*-11
	Translational initiation	1.20*E*-10
	Cytoplasmic translation	1.56*E*-10
	Translation initiation factor activity	6.93*E*-10
KEGG		
Module 1	Ribosome biogenesis in eukaryotes	2.89*E*-36
Module 2	Spliceosome	1.19*E*-15
	mRNA surveillance pathway	3.99*E*-09
	RNA transport	8.53*E*-06
Module 3	RNA transport	2.41*E*-13
	Legionellosis	0.003
	Leishmaniasis	0.005
	Spliceosome	0.02
	Ribosome	0.02

## Data Availability

This is a bioinformatic analysis tactical based on published articles. No original data were available.
